# The Acute Effect of Consuming Whey Versus a Plant‐Based Protein Blend on Postprandial Metabolism and Appetite in a Sample of Healthy Adults

**DOI:** 10.1002/fsn3.71485

**Published:** 2026-02-12

**Authors:** Yana P. Petkova, Jonathan D. Johnston, Adam L. Collins

**Affiliations:** ^1^ Department of Nutrition, Food and Exercise Sciences, Faculty of Health and Medical Sciences University of Surrey Guildford UK; ^2^ Section of Chronobiology, Faculty of Health and Medical Sciences University of Surrey Guildford UK

## Abstract

There is controversy regarding the differential metabolic effects of proteins from plant and animal sources. Existing literature focuses on a limited range of plant proteins consumed just before, or as part of, a mixed meal, resulting in limited insight into the differential effects of plant proteins when ingested alone. This study compared the acute effects of a mixed plant‐based protein supplement and whey, when ingested in a fasted state and in the absence of other nutrients, on postprandial metabolism, appetite, and subsequent energy intake. Fifteen healthy adults completed three study visits in a randomized single blind crossover study design. Participants consumed 20 g protein from whey or a plant‐based protein supplement (blend of pea, brown rice, pumpkin seeds) mixed in water, or an equal volume of water as control. Plasma ketones, non‐esterified fatty acids (NEFA), glucose, insulin, and glucagon‐like peptide 1 (GLP‐1) were measured over 3 h postprandial, alongside resting energy expenditure (REE), respiratory exchange ratio (RER), and perceived appetite. Intake from an *ad libitum* lunch meal was used to assess subsequent energy consumption. There was a transient reduction in postprandial ketones and NEFA, and elevation of insulin and GLP‐1 following protein ingestion. Compared to plant protein, whey resulted in lower NEFA at T30 (*p* = 0.037) and higher insulin concentrations at T30 (*p* < 0.001) and T60 (*p* = 0.010). REE, RER, subjective appetite ratings and subsequent energy intake were not different between the two proteins, relative to the control. The results from this study indicate that whey and a plant protein blend supplement have comparable metabolic effects.

## Graphical abstract

This study compares the metabolic effects of whey and a plant protein blend, consumed in a fasted state. The results showed a transient reduction in postprandial ketones and NEFA, and elevation of insulin and GLP‐1 following protein ingestion. The two protein drinks exerted a comparable effect on resting energy expenditure, subjective appetite ratings, and subsequent energy intake, and did not affect respiratory exchange ratio —indicating that whey and a plant protein blend supplement have comparable metabolic effects.

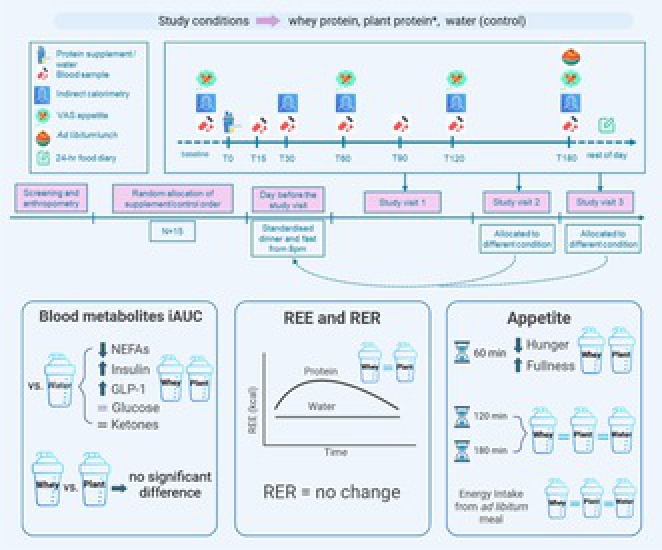

## 1 Introduction

In a time of growing academic, governmental and commercial interest in plant‐based diets for health and sustainability, understanding any differences in the metabolic effects between plant‐based and animal proteins is important (Alae‐Carew et al. 2022; Climate Change Committee 2025; Good Food Institute Europe 2024b). In the UK, similar to other countries across the globe (Abe‐Inge et al. 2024; Good Food Institute Europe 2024a), a trend for reduced intake of animal products (The Vegan Society 2025; ProVeg International 2024) is accompanied by an increased consumption of plant‐based alternative foods (Alae‐Carew et al. 2022; ProVeg International 2024). At the same time, there is growing consumer interest in higher protein diets, reflected by the increasing popularity of protein‐enriched animal‐ and plant‐based foods and protein‐related marketing claims (Maeda 2025; Butler 2025; Ocado Retail Limited 2025). In contrast to the rapidly increasing quantity of products emerging on the market, research comparing the metabolic effects of plant‐ and animal‐based proteins remains limited by a relatively small number of studies and the limited variety of plant proteins considered.

Protein from animal sources is typically regarded as higher quality compared to plant‐based proteins. The latter contain lower quantities of essential amino acids (EAA) than animal‐based protein and are often lower in leucine—an amino acid associated with muscle protein synthesis (Gorissen et al. 2018). There is, however, a notable variability in amino acid composition within the category of plant‐based proteins. For example, rice protein falls below the WHO/FAO/UNU threshold for lysine content but meets the requirements for methionine; while pea protein is low in methionine while meeting the requirements for lysine (Gorissen et al. 2018). Thus, combining different plant‐based proteins is a potential strategy to improve overall quality and ensure nutritional adequacy (Berrazaga et al. 2019).

In addition to composition, plant‐ and animal‐based proteins differ in digestibility. A higher proportion of β‐sheet conformation in the secondary structure of protein and the presence of fiber and antinutrients are thought to underlie the lower digestibility of plant proteins (Berrazaga et al. 2019). Processing, ranging from household approaches such as heating, soaking, or sprouting to treatments used in the production of commercial plant protein products, can reduce the effects of antinutrients and improve digestibility (Berrazaga et al. 2019). Thus, using combinations of protein powders from different sources has the potential to mitigate some of the perceived disadvantages of plant‐based proteins.

Consuming protein promotes diet‐induced thermogenesis, insulin and satiety hormone secretion, thus affecting postprandial energy expenditure, appetite and glycaemia (Bendtsen et al. 2013). The extent to which these metabolic effects may differ between plant and animal‐based protein sources is unclear, as research comparing the two categories has yielded mixed results (Dehnavi et al. 2023). On one hand, whey has been shown to elicit higher postprandial resting energy expenditure (REE) and satiety compared to an equivalent amount of soy protein (Acheson et al. 2011). However, others comparing whey with soy or pea protein report comparable thermic and appetite responses (Hawley et al. 2020; Melson et al. 2019). In addition to self‐reported appetite, a small number of studies have compared the effects of different types of protein on satiety hormones such as GLP‐1 but again with conflicting results (Bowen et al. 2006; Veldhorst et al. 2009; Tiekou Lorinczova et al. 2021).

Several studies comparing the glycaemic effect of protein from different sources have found that whey results in a lower postprandial glycaemic and higher insulin response compared to plant protein when part of a mixed meal (Nilsson et al. 2004; Holmer‐Jensen et al. 2013; Dougkas and Östman 2018) or served immediately before (Abou‐Samra et al. 2011; Gunnerud et al. 2012; Silva Ton et al. 2014). In contrast, Sambashivaiah et al. (2023) and Acheson et al. (2011) reported no significant difference in blood glucose responses to test meals containing whey or soy protein, despite a greater rise in postprandial insulin after whey. The conflicting findings regarding the metabolic effects of plant‐ versus animal‐based proteins are likely due to differences in study design, including the dose and timing of protein intake, as well as in test meal characteristics.

In addition to the inconclusive findings of the current literature, the research has mostly focused on a limited range of single source proteins such as soy and wheat, leaving a wide variety of plant proteins and their blends unexplored. The latter have the potential to optimize the lower EAA profile of single source plant proteins so are of particular interest. A small number of studies which have investigated the metabolic effects of plant protein blends have found comparable postprandial glucose and appetite responses with whey, but a lower postprandial aminoacidemia and mixed results regarding insulin (Dougkas and Östman 2018; Rogers et al. 2024; Pinckaers et al. 2023). Pea is emerging as a promising alternative protein source with a rapidly expanding market, but remains understudied (Hawley et al. 2020; Rogers et al. 2024). Pea and rice protein are gaining interest as their EAA profiles are complementary, resulting in a blend with a balanced EAA composition (Gorissen et al. 2018; Rogers et al. 2024; WHO/FAO/UNU 2007).

Furthermore, current literature features several methodological limitations. Firstly, the primary outcomes of interest have often been limited to postprandial aminoacidemia and muscle protein synthesis (Rogers et al. 2024; Pinckaers et al. 2023, 2024). Secondly, most existing studies compare protein from different sources as a preload served immediately before, or as part of, a mixed meal. Although this mimics free living conditions, it leaves a gap in the understanding of the metabolic effects of plant proteins when ingested on their own, for example in the form of the increasingly popular protein powder shakes.

Thus, the aim of this study was to compare the metabolic effects of a plant‐based protein blend and whey, when protein is consumed in the fasted state and in the absence of other nutrients. An important novel aspect of this study is the isolation of the independent effects of protein in triggering the transition to a postprandial state and whether these effects may be minimalised with the use of a plant protein. We hypothesized that the plant protein supplement would result in less pronounced changes to the plasma biomarkers relative to control than whey, but may have similar effects on postprandial energy expenditure and appetite.

## 2 Methods

### 2.1 Participants

Participants were recruited between February and July 2024 through advertising at the University of Surrey campus. Interested individuals were invited to complete a questionnaire assessing their eligibility to participate in the study and attend a screening session where weight and height were measured. Inclusion criteria included: age 18–45 years; BMI 18.5–27 kg/m^2^; weight stable for > 3 months; no pre‐existing metabolic condition such as diabetes, cardiovascular or eating disorder; nonsmoker; not pregnant or breastfeeding; no dietary restrictions or known allergy to ingredients used in the study; alcohol < 14 units a week; and exercise ≤ 3 times a week.

A total of 15 participants completed all three visits (nine females and six males, BMI, 22 ± 1.68 kg/m^2^; Age, 26 ± 4.44 years, mean ± SD). All participants provided written informed consent prior to starting the study. A favorable ethical opinion was obtained by the University of Surrey Ethics Committee (Ref FHMS 23‐24 022 EGA). The study was powered based on the results from Tiekou Lorinczova et al. (2021) who showed that *N* = 9 resulted in (1‐β) greater than 0.8 for GLP‐1 and insulin in a similar three‐way crossover design comparing the metabolic effects of whey, rice, and potato protein. The main difference in our protocol is the smaller serving of protein (20 g vs 50 g) and the use of water instead of low‐sugar orange juice to mix the protein powder.

### 2.2 Study Design

This study followed a single‐blind randomized controlled crossover design. Subjects attended the Human Metabolism Unit (HMU) on the university campus on three occasions, with a minimum of one week washout between visits. Participants were randomized to one of the two protein drinks (whey or plant protein) for their first study visit and to water (control) or the outstanding protein drink for their second and third study visits.

To standardize the baseline conditions, participants were provided with the same pre‐prepared meal (Spinach and Ricotta Cannelloni, Tesco) to consume by 20:00 h the evening before their study visits and instructed to fast from 20:00 h onwards, with only consumption of water allowed. Participants were also asked to abstain from exercise and alcohol for 24 h before each study visit.

**FIGURE 1:**
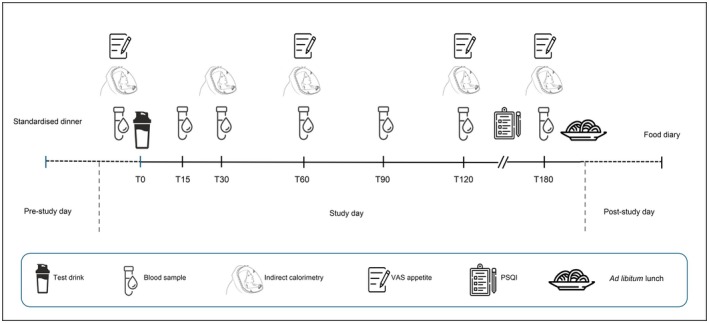
Study day timeline.

All visits followed the same structure, as outlined in Figure 1. Participants arrived at the HMU in the morning between 0800 and 0900, with exact start time consistent across the three visits. After verbal confirmation of compliance with the pre‐study day instructions, weight, resting energy expenditure (REE), respiratory exchange ratio (RER), and subjective appetite were measured. A cannula was inserted in the antecubital or basilic vein of the forearm and a baseline blood sample collected. Participants were then provided with either a protein supplement drink or equal volume of water and instructed to consume this within 10 min. Postprandial blood samples were collected at 15, 30, 60, 90, 120 and 180 min from the time participants had their first sip of the test drink. REE and RER were measured at 30, 60, 90, 120 and 180 min and subjective appetite assessed hourly using a visual analog scale (VAS) questionnaire.

After 180 min, once all measurements were obtained, participants were invited to eat as much as they would like from a lunch meal served in excess, in a room away from other participants and without a time limit. At the end of the study visit, participants were provided with a food diary and instructed to document their food and fluid intake during the rest of the day.

### 2.3 Test Drinks

Details of the nutritional composition of the test drinks can be found in Table 1. Each test drink was prepared fresh at the time of administration by mixing either 26 g of unflavoured plant protein supplement (Form Pureblend Protein) or 23 g of unflavoured whey (Bulk Whey Protein Isolate), providing 20 g protein each, with 300 mL cold water. An equal volume of water served as a control. All drinks were served in the same opaque cup throughout the study and cups were checked by the researcher to ensure the whole drink was consumed.

**TABLE 1:** Protein test drink ingredients and nutritional information including macronutrients and branched chain amino acids, per serving (26 g Pureblend protein powder and 23 g Bulk whey protein powder providing 20 g protein each).

Supplement	Form Pureblend	Bulk Pure Whey Isolate
Ingredients	Organic Pea Protein Isolate, Brown Rice Protein, Pumpkin Seed Protein	Whey Protein Isolate (Milk), Soya Lecithin
Energy (kcal)	101	83
Protein (g)	20	20
Fat (g)	1.43	0.15
Carbohydrate (g)	0	1.15
Leucine	1.95	2.17
Isoleucine	0.85	1.29
Valine	1.17	1.17

### 2.4 Anthropometric Measurements

Weight was measured at the screening visit and at the start of each study day to ensure weight stability during the study using digital scales (TANITA, NL). The same scales were used throughout the study to ensure consistency. Height was measured once during screening using a standing measure to enable BMI calculation.

### 2.5 Appetite Measures

Subjective appetite was assessed at baseline and then hourly for 3 h postprandial using a 100‐mm visual analogue score (VAS) (Flint et al. 2000), which assessed perceived hunger, fullness, and desire to eat. An *ad libitum* lunch meal consisting of pasta with a tomato and cheese sauce was served following all measures at 180 min, as a further measure of any condition‐specific effect on appetite. Detailed information on the *ad libitum* meal ingredients and nutritional composition can be found in Table S1. Participants were instructed to eat until they were comfortably full. Grams consumed were calculated by subtracting the weight of the serving bowl and leftover contents from the weight of the serving bowl and meal measured before serving, and energy intake was calculated using nutrition analysis software (Nutritics v6.04, Ireland).

Participants were also provided with a food diary at the end of each study visit to assess any further effect of the test drinks on subsequent intake. They were given verbal examples of portion sizes of common foods and instructed to record everything they had eaten and drunk for the rest of the day. The returned food diaries were analyzed using the same dietary analysis software (Nutritics v6.04).

### 2.6 Resting Energy Expenditure and Respiratory Exchange Ratio

Resting energy expenditure (REE) and respiratory exchange ratio (RER) were measured in a fasted state at the beginning of each study visit and at the specified time points during the postprandial period via gaseous exchange measured using an open circuit ventilated hood system gaseous exchange monitor (QNRQ, CosMed Ltd. Italy). To ensure that they were fully rested, participants were asked to lie supine for 15 min prior to their first measurement.

### 2.7 Plasma Metabolites

Seven blood samples of 10 mL each were collected (70 mL/study day) in lithium heparin and EDTA‐coated BD Vacutainer tubes and centrifuged at 4°C for 10 min at 2300 rpm. Plasma aliquots were stored at −80°C up to the point of analysis. Plasma glucose, ketones, and NEFA were analyzed using colorimetric assays (Randox Laboratories Ltd., UK; Thermo Fisher Scientific Inc., USA) in a clinical chemistry analyzer (Indiko Plus, Thermo Fisher Scientific Inc., USA). Insulin and total GLP‐1 concentrations were determined using enzyme‐linked immunosorbent assay kits (Merck KGaA, Darmstadt, Germany). The intra‐assay variation was 5.96% ±1.17% (Mean ± SD) and 1%–2%, and the inter‐assay variation 10.3% ±0.9% and < 10%–12% for insulin and GLP‐1, respectively.

### 2.8 Statistical Analysis

Descriptive statistics were calculated for all data and data is presented as mean and SEM unless otherwise stated. Incremental and detrimental area under the curve (iAUC and dAUC) were calculated by subtracting baseline measures from subsequent AUC readings, determined using the trapezoid method. All statistical tests were carried out in IBM SPSS (v 29.0.2.0) and statistical significance was set at *p* < 0.05. Interpolation was used to handle missing REE and RER data for one participant in the control and one participant in the whey conditions at T30.

A repeated‐measures ANCOVA, including test drink sequence as a covariate, indicated that the order in which participants received the test drinks did not affect the study outcomes. Therefore, repeated measures two‐way ANOVAs with *post hoc* Bonferroni correction were used to analyze differences over time in plasma biomarkers, REE, RER, and subjective appetite between the study conditions. One‐way repeated measures ANOVAs with *post hoc* Bonferroni correction were used for analysis of the *ad libitum* and rest of day energy intake, as well as for iAUC and the PSQI scores.

## 3 Results

Fasting measures for all variables and mean PSQI scores were not different between the three study conditions as assessed using one‐way repeated ANOVAs.

### 3.1 Biochemistry

**FIGURE 2:**
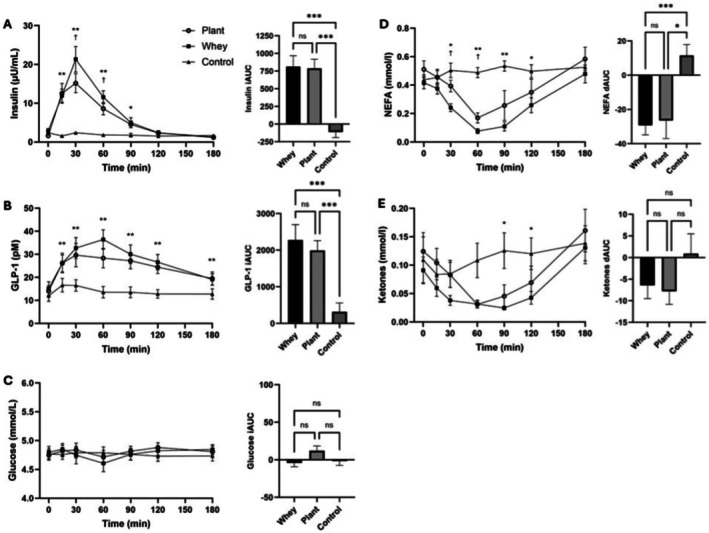
Three‐hour postprandial concentrations and incremental/detrimental area under the curve (iAUC/dAUC) of plasma insulin (A), glucagon‐like peptide 1 (GLP‐1) (B), glucose (C), non‐esterified fatty acids (NEFA) (D) and ketones (E); N=15 Results expressed as mean (±SEM). Line graph: *protein different from control (*P*<.0.05), **both proteins different from control (*P* < 0.05), ^†^plant protein different from whey (*P* <.0.05). Bar chart: *P < 0.05, ***P < 0.001.

The changes in plasma biomarkers in the three‐hour postprandial period are illustrated in Figure 2. There was a significant effect of condition and time, and a condition × time interaction for insulin and GLP‐1 concentrations (all *p* < 0.001). Postprandial insulin concentrations increased after intake of whey and plant protein, peaking at 60 min postprandial. The magnitude of insulin increase was greater after whey at T30 (*p* < 0.001) and T60 (*p* = 0.010) compared to plant protein. Insulin concentration after intake of either protein drink was not significantly different from the water‐only arm at T120 and T180.

There was a rise in GLP‐1 concentrations within 15 min postprandial after both whey and plant protein relative to water (*p* < 0.001 and *p* = 0.006, respectively). GLP‐1 remained significantly higher after whey and plant protein throughout the 3 h postprandial (all *p* < 0.05), albeit with no significant difference between whey and plant protein at any timepoint. There was no effect of condition or time on glucose concentrations, and no condition × time interaction (all *p* > 0.05).

Mean insulin and GLP‐1 iAUC were greater in the whey and plant protein treatments compared to control (both *p* < 0.001), with no significant difference between the two protein sources. There was no significant difference in glucose iAUC between the three study conditions.

Postprandial NEFA concentrations after both protein drinks decreased and were at their nadir at T60, while ketone concentrations were at their lowest at T60 in the whey and at T90 in the plant condition.

There was a significant effect of condition (*p* = 0.001) and time (*p* < 0.001) on NEFA concentrations, and a condition × time interaction (*p* < 0.001). NEFA concentrations were significantly lower after whey compared to plant protein (*p* = 0.037) and water (*p* = 0.003) at T30, and lower after both protein drinks at T60 (both *p* < 0.001) and T90 (*p* < 0.001 for whey and *p* = 0.048 for plant protein). At 2 h postprandial, NEFA concentrations remained lower relative to water in the whey arm only (*p* = 0.011). Whey resulted in a greater reduction in NEFA concentrations than plant protein at T30 (*p* = 0.037) and T60 (*p* = 0.01).

There was a main effect of time and a condition × time interaction on ketones (both *p* < 0.001). Ketone concentrations were lower in the whey compared to the control arm at T90 (*p* = 0.017) and T120 (*p* = 0.031). There were no other significant differences between the study arms across the measurement time points. Both NEFA and ketone concentrations were not significantly different from the water‐only arm by T180.

NEFA dAUC was lower in both the whey (*p* < 0.001) and plant protein (*p* = 0.011) treatments compared to control, with no significant difference between the two proteins. There was no significant difference in ketone dAUC between the study conditions.

### 3.2 REE and RER

**FIGURE 3:**
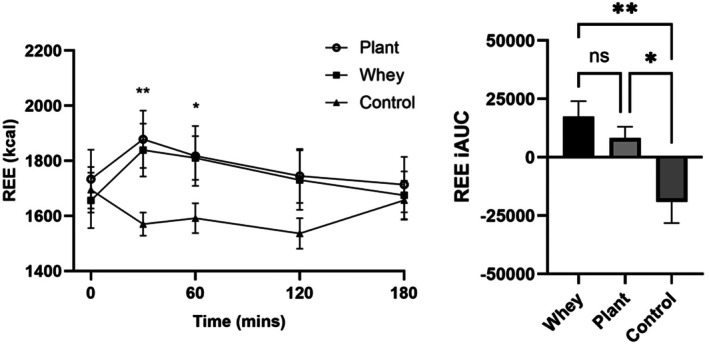
Postprandial REE (kcal) and iAUC following ingestion of whey, an (unsweetened) plant protein supplement and water; mean (±SEM). *Protein different from control (*p* < 0.0.05), **both proteins different from control (*P* < 0.05).

There was a significant effect of condition (*p* = 0.012) and time (*p* = 0.021) on REE, and a condition × time interaction (*p* < 0.001; Figure 3). REE was significantly higher in the whey and plant protein arms compared to control at T30 (*p* = 0.018 and *p* = 0.013, respectively) and in the whey arm only at T60 (*p* = 0.008). This was reflected in REE iAUC, which was greater after both protein treatments than control (*p* = 0.006 whey, *p* = 0.032 plant protein). There was an effect of time on RER (*p* < 0.001), with mean RER significantly lower compared to baseline at 2 h and 3 h postprandial (*p* = 0.024 and *p* < 0.001) (Table 2). There was no effect of condition and no condition × time interaction.

**TABLE 2:** Respiratory exchange ratio (RER) at baseline and over 3 h postprandial, data presented as mean (SEM).

Time (mins)	Plant protein, Mean (SD)	Whey, Mean (SD)	Water, Mean (SD)
0	0.78 (0.01)	0.79 (0.01)	0.80 (0.01)
30	0.78 (0.01)	0.78 (0.01)	0.78 (0.01)
60	0.79 (0.01)	0.77 (0.01)	0.77 (0.01)
120	0.76 (0.01)	0.77 (0.01)	0.77 (0.01)
180	0.76 (0.01)	0.75 (0.01)	0.77 (0.01)

### 3.3 Appetite and Energy Intake

**FIGURE 4:**
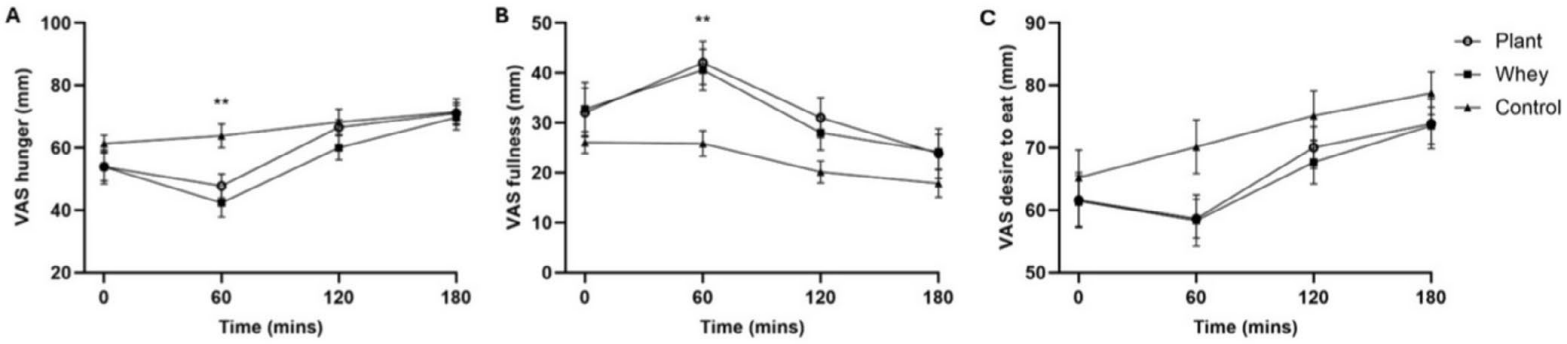
Perceived hunger, fullness and desire to eat at baseline and hourly for 3 h postprandial following ingestion of whey, a plant protein supplement and water; mean (±SEM). * protein different from control (*p* < 0.0.05), ** both proteins different from control (*P* < 0.05).

Subjective appetite ratings assessed through a VAS questionnaire are illustrated in Figure 4. There was an effect of condition (*p* = 0.013) and time (*p* < 0.001) on perceived hunger, and a condition × time interaction (*p* = 0.034). Hunger ratings were significantly lower in both the whey (*p* = 0.003) and plant protein (*p* = 0.021) arms relative to control at T60. At 2 h‐ and 3 h postprandial, perceived hunger was not significantly different between the protein and water‐only conditions. There was an effect of condition (*p* = 0.007) and time (*p* < 0.001) on perceived fullness. Feelings of fullness were greater after both protein drinks compared to control at T60 (*p* = 0.008 after whey and *p* = 0.046 after plant protein). There was an effect of condition (*p* = 0.018) and time (*p* < 0.001) on desire to eat, which was lower after whey compared to the control arm (*p* = 0.024), with no significant difference between plant protein and control or between the two protein sources. Mean desire to eat increased over time and was significantly higher at T20 and T180 compared to baseline (*p* = 0.022 and *p* = 0.003, respectively).

Mean (±SEM) energy intake from the *ad libitum* lunch meal was 1275 kcal (±132 kcal), 1309 kcal (±153 kcal), and 1425 kcal (±140 kcal) in the plant protein, whey, and control conditions respectively. There was no significant difference between the three conditions. Similarly, there was no effect of condition on energy intake for the rest of the day in *N* = 13 participants who completed all 24‐h food diaries.

## 4 Discussion

Existing research comparing the metabolic effects of protein from animal and plant‐based sources remains limited by a relatively small, albeit increasing, number of studies with conflicting findings, and a narrow range of plant proteins examined. To our knowledge, this is the first study to compare the effects of whey and a plant protein blend, ingested in the absence of other nutrients, on plasma biomarkers of fasting metabolism, REE, RER, and appetite.

Protein from animal sources generally has a higher concentration of insulinogenic branched chain amino acids (BCAAs) such as leucine, isoleucine, and arginine than plant proteins (Berrazaga et al. 2019). Whey, in particular, has a high concentration of leucine and has been shown to have a greater insulinemic effect than protein from other sources such as soy, gluten, cod, casein, and milk (Acheson et al. 2011; Nilsson et al. 2004), although some studies have failed to replicate this effect (Dougkas and Östman 2018; Mortensen et al. 2009) likely due to differences in study design such as different study populations and macronutrient composition of the test meals.

One possible explanation for the greater rise in postprandial insulin following whey observed in the present study is the higher concentrations of insulinotropic BCAAs in the whey supplement compared to the plant protein, despite the plant protein mixture providing a full complement of EAAs. Although postprandial plasma amino acids were not measured in the present protocol, others have demonstrated higher concentrations of plasma BCAAs following meals with whey compared to plant‐based proteins (Acheson et al. 2011; Nilsson et al. 2004; Rogers et al. 2024). It should be noted that the whey protein powder contained a small amount of carbohydrate (1.15 g) while the plant protein supplement contained none. Whilst this may have been a confounding factor, it is unclear whether such a negligible amount is enough to elicit a measurable effect on the insulin response given that carbohydrate doses administered in existing metabolic studies are significantly higher.

The difference in insulin elevation may explain the greater magnitude of change in postprandial NEFA and ketone concentrations we observed following whey, compared to plant protein. Fasting for > 12 h causes a metabolic shift from glycogenolysis towards gluconeogenesis and fat oxidation—a state characterized by a rise in plasma NEFAs and ketones, and low levels of insulin and GLP‐1 (Frayn 2019; Monnier 2000). One of the many functions of insulin is to suppress lipolysis; thus, a greater rise in plasma levels may induce a greater suppression in NEFA and ketone production.

It should be noted that the plant protein supplement contained a small amount of fat (1.43 g). Fat intake has been shown to contribute to the NEFA pool through spillover from lipolysis of TAG in chylomicrons by lipoprotein lipase (LPL) (Frayn 2019). Thus, the discrepancy in fat composition between the two protein drinks could pose a confounding factor and is another possible explanation for the differences in postprandial NEFA concentration at T30 and T60. Whether such a negligible amount of fat as that in the plant protein supplement is enough to have a measurable effect on plasma NEFA is unclear. The lack of difference between NEFA dAUC in the two protein treatments legs suggests that any such effect is likely to be minimal.

Interestingly, the transient reduction in ketone and NEFA concentrations following protein ingestion was not reflected in a change in RER from baseline or a difference in RER between conditions. It is possible that such subtle changes in substrate utilization, at the tissue level, were too small to be detected through indirect calorimetry. Existing studies comparing animal and plant proteins report no difference in postprandial substrate utilization (Acheson et al. 2011; Hawley et al. 2020; Melson et al. 2019; Tan et al. 2010). Our study's uniqueness is in the comparison of the metabolic effects of whey and a plant protein ingested in the absence of other nutrients. Whilst our findings support those reported previously, we additionally demonstrated that although whey may cause a more pronounced acute shift in fasting metabolism compared to a mixed plant protein, the magnitude of these changes might not be enough to “flip the metabolic switch” back to glucose utilization for energy (Anton et al. 2018).

Despite the subtle differences in plasma metabolites, we observed similar appetite responses between the two protein conditions, in line with existing literature (Hawley et al. 2020; Rogers et al. 2024; Tan et al. 2010; Crowder et al. 2016). Both whey and plant protein, when ingested in the absence of other macronutrients, reduced hunger and increased feelings of fullness in the first hour after intake. This is in line with the observed rise in GLP‐1 following protein intake but not water. It has been suggested that proteins from plant and animal sources may stimulate GLP‐1 secretion to a different extent (Tiekou Lorinczova et al. 2021). However, we observed comparable changes in postprandial GLP‐1 concentrations between the whey and plant protein conditions. Similarly, Rogers et al. (2024) reported no significant difference in postprandial GLP‐1 AUC following 20 g of pea and whey protein. The lack of protein‐specific effect may be a consequence of the relatively small quantity of protein used in our study, as previous research indicates a possible dose–response effect with a higher protein intake necessary to elicit a difference in GLP‐1 (Veldhorst et al. 2009). Furthermore, the present study was not powered based on GLP‐1.

Several mechanisms have been proposed to explain the thermic effect of protein including the energetic cost of amino acid oxidation, urea synthesis, and postprandial muscle protein synthesis (MPS) (Dehnavi et al. 2023; Acheson et al. 2011). Body protein synthesis has been reported to occur within 2 h following protein intake (Groen et al. 2015; Gorissen et al. 2015). Existing research on the impact of protein from animal and plant‐based sources on energy expenditure has yielded mixed results (Acheson et al. 2011; Hawley et al. 2020; Melson et al. 2019; Mikkelsen et al. 2000). In line with findings from Melson et al. (Melson et al. 2019) and Hawley et al. (Hawley et al. 2020) we observed similar trends in postprandial REE following ingestion of 20 g of whey and plant protein. In a series of recent studies, Pinckaers and colleagues have demonstrated that plant proteins can stimulate MPS to a comparable extent as milk protein, despite an overall lower content of EAA (Pinckaers et al. 2023, 2024, 2021). Moreover, processing such as is used in the production of commercial protein powders has been shown to improve plant protein digestibility, eliminating another potential contributor to a difference in REE (Nichele et al. 2022).

### 4.1 Limitations

The present study has several limitations. Due to budgetary constraints, we were unable to measure digestion kinetics and amino acid appearance in the blood. Others have compared postprandial plasma amino acid concentrations following meals with plant or animal proteins and found higher concentrations of plasma BCAAs following whey compared to soy (Acheson et al. 2011; Veldhorst et al. 2009) and gluten (Nilsson et al. 2004). However, these are not directly applicable due to the different composition of the plant proteins used in previous and the current protocols.

We did not control for palatability of the test drinks, a factor which may affect appetite (Johnson and Wardle 2014). The lack of a difference between the two protein conditions suggests that palatability may have been comparable or not have been a factor in this instance. Finally, beyond scheduling study visits to avoid menstruation, we did not control for menstrual cycle phase, which may affect gastric emptying and thus postprandial metabolic response (Brennan et al. 2009). With regards to insulin sensitivity and glucose metabolism, results from existing research have been controversial (Schieren et al. 2024).

### 4.2 Conclusion and Future Research

In summary, our study demonstrated that consuming 20 g protein after a 12‐h overnight fast led to significant but small increases in plasma insulin and GLP‐1 and a decrease in NEFA and ketones, with no effect on blood glucose, and that some of these changes were greater after whey compared to plant protein. The shift from fasting metabolism indicated by the changes in these blood metabolites and hormones, however, was not reflected by a difference in RER—suggesting that 20 g plant protein, in the absence of other nutrients, may not be enough to alter substrate utilization akin to a metabolic transition from the fasted to a postprandial state. Moreover, the present study suggests that whey and plant protein elicit a comparable appetite response.

The present results may pave the way for future research on the potential of allowing the consumption of plant protein, in the absence of other macronutrients, within the confines of fasting protocols such as time‐restricted eating. Such an approach may help optimize protein intake in populations with higher needs while maintaining some of the benefits of fasting.

## Author Contributions

**Yana P. Petkova:** conceptualization (equal), formal analysis (lead), investigation (lead), methodology (equal), project administration (lead), writing – original draft (lead). **Adam L. Collins:** conceptualization (equal), formal analysis (supporting), funding acquisition (lead), methodology (equal), supervision (equal), writing – original draft (supporting). **Jonathan D. Johnston:** supervision (supporting), writing – original draft (supporting).

## Funding

This work was supported by UKRI BBSRC FoodBioSystems Doctoral Training Partnership, BB/T008776/1.

## Conflicts of Interest

Dr. A.L. Collins serves as a consultant for the commercial company FormNutrition Ltd., who supplied the plant‐based protein product used in the study. FormNutrition Ltd. was not involved in the study design, data collection, analysis and manuscript preparation.

## Supporting information


**Table S1:** Ad libitum lunch meal recipe and nutritional composition; whole recipe served to participants.

## Data Availability

The data that support the findings of this study are available from the corresponding author upon reasonable request.
