# Supplementary material for: The Acute Effect of Consuming Whey Versus a Plant‐Based Protein Blend on Postprandial Metabolism and Appetite in a Sample of Healthy Adults
Source: Food Sci Nutr. 2026 Feb 12;14(2):e71485. doi: 10.1002/fsn3.71485 (PMC12900885; doi:10.1002/fsn3.71485)
Supplement: Supplementary file 1 — Table S1: Ad libitum lunch meal recipe and nutritional composition; whole recipe served to participants. [file FSN3-14-e71485-s001.docx]

**Table S1.** Ad libitum lunch meal recipe and nutritional composition; whole recipe served to participants

| Ingredients | Weight |
| --- | --- |
| Dry weight fusilli pasta (Tesco) | 400g |
| no added sugar tomato sauce (Tesco) | 500g |
| mature cheddar cheese (Lidl) | 100 g |
| vegetable oil (Tesco) | 30 ml |
| Nutritional information per 100g |  |
| Energy (kcal) | 220 |
| Protein (g) | 7.8 |
| Fat (g) | 7.3 |
| Carbohydrate (g) | 30 |
| Fibre (g) | 2.4 |
